# A Molecular Survey of *Babesia* Species and Detection of a New *Babesia* Species by DNA Related to *B. venatorum* from White Yaks in Tianzhu, China

**DOI:** 10.3389/fmicb.2017.00419

**Published:** 2017-03-14

**Authors:** Junlong Liu, Guiquan Guan, Youquan Li, Aihong Liu, Jianxun Luo, Hong Yin

**Affiliations:** ^1^State Key Laboratory of Veterinary Etiological Biology, Key Laboratory of Veterinary Parasitology of Gansu Province, Lanzhou Veterinary Research Institute, Chinese Academy of Agricultural SciencesLanzhou, China; ^2^Jiangsu Co-innovation Center for Prevention and Control of Important Animal Infectious Diseases and ZoonosesYangzhou, China

**Keywords:** white yak, *Babesia*, *Babesia venatorum*, 18S rRNA, Northwest China

## Abstract

Bovine babesiosis is a tick-transmitted disease caused by different species of *Babesia*. The white yak is a unique yak breed that lives only in Tianzhu in the Tibetan Autonomous County, Gansu Province, in northwestern China. Previous research using the ELISA method has confirmed that the white yak could become infected with *B. bigemina*. The objective of this study was the molecular detection and identification of *Babesia* species in white yaks. A total of 409 white yak blood samples were collected from 11 areas of the Tianzhu Tibetan Autonomous County in Northwest China from April to August, 2015. The V4 hypervariable region of *Babesia* 18S rRNA was amplified from extracted genomic DNA using nested PCR and sequenced. The nearly full-length sequence of 18S rRNA including the V4 region from the newly discovered *Babesia* was amplified and sequenced with Sanger method. PCR detection and sequencing indicated that 4/409 samples were positive for *B. bigemina*, 3/409 samples were positive for *B. bovis*, and 5/409 samples were positive for *B. ovata.* Additionally, a new *Babesia* species was found in 4/409 white yaks. A unique sequence of 1,627 bp was obtained from two of the four samples. The sequence was similar to *Babesia* species Akita (98.5%) found in *Ixodes ovatus* and *B. venatorum* (98%) and shared a 98% identity with *B. divergens* and a 98.1% identity with *B. odocoilei*. This study provides new data about *Babesia* infections in white yaks in northwestern China, and a new *Babesia* species similar to *B. venatorum* was identified in white yaks for the first time.

## Introduction

Bovine babesiosis is a tick-borne disease caused by the intra-erythrocytic apicomplexan protozoan parasites of the different species of *Babesia*. *B. bovis* and *B. bigemina* are the most common bovine babesiosis pathogens and have an important economic and veterinary impact in ruminants (Kim et al., 2008). These and other *Babesia* species, including *B. ovata, B. major, B. orientalis*, and *Babesia* sp. Kashi, have been confirmed to exist in China (Yin et al., 1997; Luo et al., 2002; Liu et al., 2008). In addition, some *Babesia* species can infect both humans and animals, one of which is *B. divergens*, the primary agent of human babesiosis in Europe (Malandrin et al., 2010). In China, two patients from Shandong Province were confirmed to be infected with *B. divergens* (Qi et al., 2011), but *B. divergens* infections in cattle have not been reported in China until now.

The white yak (*Bos grunniens*) is a special yak breed that mainly lives in Tianzhu in the Tibetan Autonomous County (TTAC), Gansu Province, in northwestern China (Qin et al., 2014). Because TTAC is part of the Qinghai-Tibet Plateau where the weather is dry and cold, white yaks are important to the local residents for things such as meat and milk production. White yaks are considered semi-wild animals in China and thus have more random contact with ticks, which could transmit *Babesia*. *B. bigemina* is a parasite that can infect cattle and buffalo and was recently confirmed to infect black yaks and white yaks (He et al., 2012; Schnittger et al., 2012; Saravanan et al., 2013; Qin et al., 2015). Additionally, some black yak blood samples from Gannan in the Tibetan Autonomous County were confirmed to be infected with *B. bovis* by PCR (Niu et al., 2015). However, no systematic data concerning *Babesia* infections in white yaks have been reported until now. In this study, blood samples from white yaks were collected from 11 areas of the TTAC to determine which species of *Babesia* might have infected white yaks.

## Materials and Methods

### Blood Samples

A total of 409 blood samples were randomly collected from white yaks from 11 areas of the TTAC (**Table 1**). Blood samples from the jugular vein were collected in tubes containing EDTA and then stored at 4°C until DNA extraction. The procedure of the samples collection was similar as described in the paper for detection of *Babesia* and *Theileria* (Lempereur et al., 2017).

**Table 1:** The number of positive blood samples for *Babesia* spp. infection from white yaks from Tianzhu Tibetan Autonomous County, Gansu province.

Location	Total no.	*B. bigemina*	*B. bovis*	*B. ovata*	*B.* sp
Saisishi	40	0	1	0	0
Tiantang	53	3	2	1	1
Maozang	59	0	0	2	3
Dongdatan	41	1	0	2	0
Anyuan	20	0	0	0	0
Xidatan	25	0	0	0	0
Shimen	30	0	0	0	0
Zhuaxixiulong	31	0	0	0	0
Duoshi	30	0	0	0	0
Tanshancen	30	0	0	0	0
Danma	50	0	0	0	0
Total number	409	4	3	5	4

### DNA Extraction

DNA was extracted from a 300-μL aliquot of each blood sample with a Genomic DNA Purification Kit by following the manufacturer’s instructions (Qiagen, Hilden, Germany). The extracted DNA was eluted in 100 μL of elution buffer, and the concentration was determined with a NanoDrop 2000 spectrophotometer (NanoDrop Technologies, Wilmington, DE, USA). The DNA was stored at -20°C until further analysis.

### PCR Amplification of the V4 Region of the 18S rRNA Gene

Nested PCR was used to amplify the V4 hypervariable region of the 18S rRNA of *Babesia* using previously reported universal primers (Gubbels et al., 1999; Centeno-Lima et al., 2003; Zanet et al., 2014; Liu et al., 2016). The first PCR reaction was conducted with the primers RLB-F2 (5′-GACACAGGGAGGTAGTGACAAG-3′) and RLB-R2 (5′-CTAAGAATTTCACCTCTGACAGT-3′) in a 25 μL total volume containing 12.5 μL Premix Taq DNA Polymerase (TaKaRa, Dalian, China), 1 μM of each primer and 1 μL of genomic DNA. The PCR reaction was started with a one-step initial denaturation at 95°C for 3 min, which was followed by 35 cycles of denaturation at 95°C for 1 min, 52°C for 50 s and extension at 72°C for 1 min, with a final extension at 72°C for 5 min. The primers RLB-FINT (5′-GACAAGAAATAACAATACRGGGC-3′) and RLB-R2 were, respectively, used as the forward and reverse primers in a second PCR. The reaction mixture had the same composition as in the first PCR, except that the template was replaced by 1 μL of the first PCR product. The reaction cycling comprised an initial denaturation step of 95°C for 3 min, followed by 35 cycles of denaturation (95°C for 30 s), primer annealing (50°C for 30 s) and extension (72°C for 30 s). A final extension was performed at 72°C for 5 min. Each PCR product was electrophoresed on a 1.5% agarose gel containing 10 μL of gold view dye (SolarBio, Tianjin, China) in Tris-acetate-EDTA (TAE) buffer at 120 V for 30 min and visualized under UV light.

The positive PCR products were cloned into the pGEM-T Easy vectors (Promega, USA) and transformed into *Escherichia coli* JM109 (TaKaRa, China). At least five positive clones were sequenced using an ABI Prism Terminator Cycle Sequencing kit and carried out on an Applied Biosystem 3730 DNA Analyzer (Sangon Biotech, Shanghai, China) to obtain consensus sequences.

### PCR Amplification of the Long Fragment of 18S rRNA

A long sequence of 18S rRNA which containing the V4 region was amplified with nested PCR, to clarify the classification of the newly discovered *Babesia*. The first round PCR was amplified using the primer set PiroF (5′-GCCAGTAGTCATATGCTTGTGTTA-3′) and Piro6R (5′-CTCCTTCCTYTAAGTGATAAGGTTCAC-3′). Another pair of primers, Piro1F (5′-CCATGCAGTTCTWAGTAYAARCTTTTA-3′) and Piro5.5R (5′-CCTYTAAGTGATAAGGTTCACAAAACTT-3′) were used in the second PCR (Kawabuchi et al., 2005). The composition of the PCR reactions was the same described as above. The PCR reactions were initiated with one step of 95°C for 3 min, followed by 35 cycles of denaturation (94°C for 1 min), primer annealing (59°C for 1 min) and extension (72°C for 2 min). A final extension was performed at 72°C for 10 min. The cloning and sequencing procedure was the same as described above.

### Sequence Analysis

The sequences obtained were aligned with the related *Babesia* sp. 18S rRNA by using the MegAlign component of the DNAStar software program (Version 4.0 DNAStar, Madison, WI, USA). The section containing the cloning vector sequence was manually removed. The final 18S rRNA sequences were submitted to the GenBank database.

A phylogenetic analysis of the 18S rRNA genes determined in this study with other sequences registered in GenBank was carried out with MEGA 6.0 software. The distance matrices for the aligned sequences were calculated by the Kimura two-parameter method, and the neighbor-joining method was used to generate a phylogenetic tree (Kimura, 1980; Tamura et al., 2013).

### Ethical Approval

The present work was approved by the Animal Ethics Committee of Lanzhou Veterinary Research Institute CAAS (No. LVRIAEC2013-010). The procedures for acquiring the field samples were in accordance with the Animal Ethics Procedures and Guidelines of China.

## Results

**FIGURE 1:**
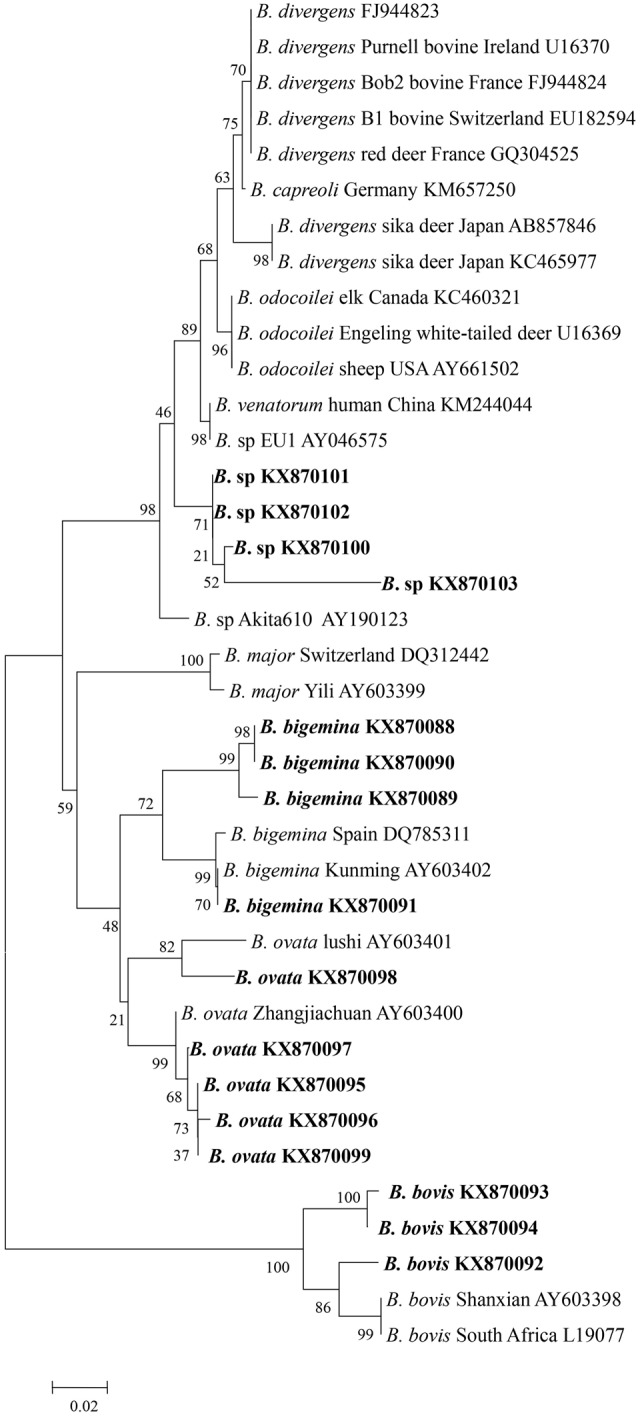
**Phylogenetic tree of *Babesia* spp. based on the V4 region of 18S rRNA gene sequences.** The parasites identified in the present study are marked in bold.

Genomic DNA was successfully extracted from the blood samples, and the positive PCR products that targeted the 18S rRNA V4 region of the piroplasms were sequenced. The sequences were then phylogenetically analyzed with related *Babesia* species 18S rRNA gene sequences deposited in GenBank. The analysis of results identified four samples from Tiantang and Dongdatan as *B. bigemina* (GenBank accession no. KX870088, KX870089, KX870090, and KX870091). The other three samples from Saisishi and Tiantang were grouped with *B. bovis* (GenBank accession no. KX870092, KX870093 and KX970094). The five samples from Tiantang, Maozang and Dongdatan, were identical with *B. ovata* (GenBank accession no. KX870095, KX870096, KX870097, KX870098, and KX870099). The four samples from Tiantang and Maozang were closely related to *B. venatorum, B. odocoilei*, and *B.* sp Akita that was identified in *Ixodes ovatus* from Japan (GenBank accession no. KX870100, KX870101, KX870102, and KX870103) (**Table 1** and **Figure 1**). No co-infections were observed in the samples analyzed.

**FIGURE 2:**
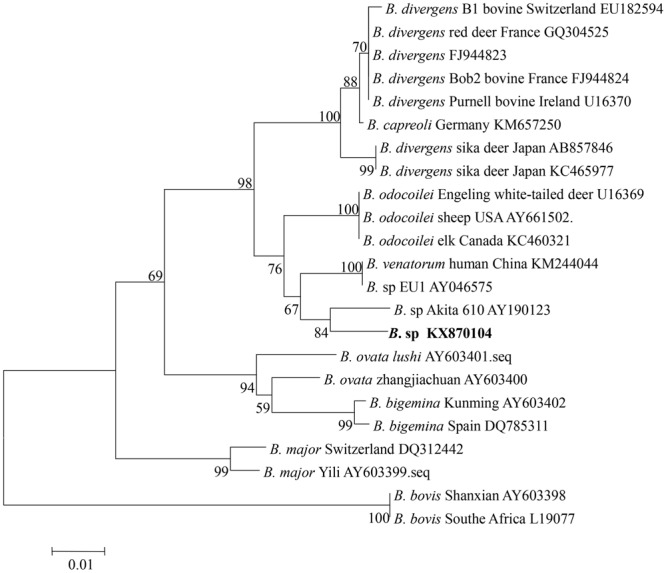
**Phylogenetic relationship of various *Babesia* spp. based on the nucleotide sequences of the 18S rRNA gene.** The parasite identified in the present study is marked in bold.

To confirm the classification of the newly discovered *Babesia* species that was identified based on the V4 region of 18S rRNA, a nearly full-length 18S rRNA gene sequence of *Babesia* was amplified from the four samples from Tiantang and Maozang. Only two samples from Maozang produced a fragment of approximately 1700 bp. After removal of the vector sequence, an identical sequence which contained 1672 bp (GenBank accession no. KX870104) was obtained from those two samples. The sequence shared a 98.5% identity with *B. sp.* Akita, a 98.1% identity with *B. odocoilei*, and a 98% identity with *B. venatorum* and *B. divergens*. The phylogenetic analysis showed that the newly discovered *Babesia* from white yak belonged to the group containing *B. divergens, B. odocoilei, B. venatorum*, and *B.* sp Akita (**Figure 2**).

## Discussion

In the present study, a total of 409 blood samples from white yaks was collected and tested for *Babesia* infection. Three species of *Babesia* previously reported in China were identified, including *B. bovis, B. bigemina*, and *B. ovata*. Because they share the same transmission vector (*Rhipicephalus microplus*), *B. bovis*, and *B. bigemina* often co-infect a host. The area with the greatest prevalence of those two parasites is central and southern China, and the primary host is cattle (Liu et al., 2014). In addition to cattle, water buffalo has also been confirmed to be infected with *B. bovis* and *B. bigemina* by both DNA and serum detection methods (He et al., 2012). A few reports have described the infection of *B. bigemina* in yaks including black and white yaks in China and Nepal by using serological methods (Graves et al., 1975; Qin et al., 2015). Recent studies have confirmed *B. bigemina* infection in yaks with PCR targeting the 18S rRNA fragment of *B. bigemina* and by further sequencing analysis as well as restriction digestion of a PCR amplified fragment (Saravanan et al., 2013). In the present study, four white yak samples tested positive for *B. bigemina* genomic DNA. A specific PCR method for amplifying the rhoptry-associated-protein-1 gene was used to detect *B. bovis* in black yaks from the Gannan Tibetan Autonomous Prefecture (GTAP), Gansu Province, which first confirmed the *B. bovis* infection of black yaks. In the present study, three white yaks from the TTAC, which has similar environmental conditions to the GTAP, were identified as infected with *B. bovis*. This study is the first report of the *B. bovis* infection of white yaks from the TTAC.

*Babesia ovata* is mainly transmitted by *Haemaphysalis longicornis* in China, and it was confirmed to exist in Gansu, Henan, and Sichuan Provinces (Liu et al., 2008). The vertebrate host for *B. ovata* is cattle, and no other animals have been reported as hosts. *B. ovata* infection was identified in white yaks in this study. Five blood samples from three areas of the TTAC were confirmed as *B. ovata* positive by sequencing analysis. During blood sample collection, ticks were also collected from both the grass and the yaks, and two species (*H. qinghaiensis* and *H. longicornis*) were identified, indicating the possibility of *B. ovata* transmission in the TTAC.

The phylogenetic analysis grouped *Babesia* 18S rRNA gene nucleotide sequences from four samples from Tiantang and Maozang with the normal bovine *Babesia* species but also showed a close relationship with *B. venatorum* and *Babesia* species Akita 610. These results suggest that the discovered *Babesia* species might be a new *Babesia* found in bovines and is the first time that this group of *Babesia* has been detected in white yaks from Gansu Province. *B. venatorum*, previously named *Babesia* sp. EU1, was first reported in two asplenic men from Italy and Austria, and later, a 63-year-old man from Germany was confirmed as infected with *B. venatorum* (Herwaldt et al., 2003; Häselbarth et al., 2007). This parasite was also identified in reindeer, and *I. ricinus* was confirmed as the transmission vector (Bonnet et al., 2009; Kik et al., 2011). A recent study has indicated the high prevalence of *B. venatorum* in *I. persulcatus*, suggesting that this tick might an important vector for *B. venatorum* in Mongolia (Karnath et al., 2016). *B. venatorum* was also found to infect humans in China. In 2012, a 8-year-old child from the Xinjiang Autonomous Region was confirmed as infected with *B. venatorum* (Sun et al., 2014). Another study described 48 cases, including 32 confirmed cases and 16 probable cases from Heilongjiang Province, of *B. venatorum* infections. In the same study, *B. venatorum* DNA was also detected in *I. persulcatus* (Jiang et al., 2015). In Japan, a *Babesia* species Akita610 closely related to *B. venatorum* was found in *I. ovatus* collected from dogs, but *Babesia* species were not detected in dogs (Inokuma et al., 2003). The tick vectors of *B. venatorum* were not found in the area where the samples were collected for our study. However, in the Gannan Tibetan Autonomous Prefecture, which has a similar climate as the TTAC, a few *I. ovatus* have been collected (personal communication). Thus, the details of the tick distribution in the TTAC should be determined to clarify the vector for the newly discovered *Babesia*. Moreover, epidemiological studies of humans infected with this *Babesia* species are necessary because *B. venatorum* is known as a human pathogen and several cases of *B. venatorum* infection in humans have been reported in European countries and in China.

## Conclusion

In this study, a total of 409 white yak blood samples from the TTAC were used for an epidemiological study of *Babesia* infection. Three normal bovine *Babesia* species including *B. bigemina, B. bovis*, and *B. ovata* were detected in white yaks, and based on 18S rRNA gene analysis, a new *Babesia* species closely related to *B. venatorum* and *B. sp* Akita was found in four white yaks. The tick vector (*H. longicornis*) for *B. ovata* was found in this area, but the vectors for *B. bovis, B. bigemina*, and *B. venatorum* were not found. Thus, more studies must be carried out to study the distribution of tick species in this area and to elucidate the life cycle of *B. bovis, B. bigemina* and the newly discovered *Babesia*.

## Author Contributions

JLL, GG, and AL did the sample collection and the molecular genetic studies. JLL wrote the draft of the manuscript. GG, YL, JXL, and HY corrected the manuscript. All authors read and approved the final manuscript.

## Conflict of Interest Statement

The authors declare that the research was conducted in the absence of any commercial or financial relationships that could be construed as a potential conflict of interest.
